# MUSE and PROPELLER DWI for ADC in parasagittal dura: insights from high-resolution and reduced-distortion DWI

**DOI:** 10.1038/s41598-025-91751-0

**Published:** 2025-03-03

**Authors:** Yi-Jui Liu, Shao-Chieh Lin, Chun-Han Liao, Shin-Lei Peng, Yi-Xian Lu, Chi-Feng Hsieh, Chiao-Hua Lee, Ming-Ting Tsai, Chun-Jung Juan, Ya-Hui Li, Hing-Chiu Chang

**Affiliations:** 1 Department of Automatic Control Engineering, Feng Chia University, Taichung, 407 Taiwan, Republic of China; 2 Ph.D. Program in Electrical and Communication Engineering, Feng Chia University, Taichung, 407 Taiwan, Republic of China; 3 Department of Medical Imaging, Changhua Christian Medical Foundation Yuanlin Christian Hospital, Changhua, 510 Taiwan, Republic of China; 4 Department of Medical Imaging, Changhua Christian Hospital, Changhua, 500 Taiwan, Republic of China; 5 Department of Biomedical Imaging and Radiological Science, China Medical University, Taichung, 404 Taiwan, Republic of China; 6 Master’s Program of Biomedical Informatics and Biomedical Engineering, Feng Chia University, Taichung, 407 Taiwan, Republic of China; 7 Department of Medical Imaging, China Medical University Hsinchu Hospital, No. 199, Sec. 1, Xinglong Rd., Zhubei City, 302 Hsinchu County Taiwan, Republic of China; 8 Department of Radiology, School of Medicine, China Medical University, Taichung, 404 Taiwan, Republic of China; 9 Department of Medical Imaging, China Medical University Hospital, Taichung, 404 Taiwan, Republic of China; 10 Department of Biomedical Engineering and Environmental Sciences, National Tsing Hua University, Hsinchu, 300 Taiwan, Republic of China; 11 Graduate Institute of Biomedical Electronics and Bioinformatics, National Taiwan University, Taipei, Taiwan, Republic of China; 12 Department of Biomedical Engineering, The Chinese University of Hong Kong, Room 1112, 11/F, William M.W. Mong Engineering Building, Shatin, N.T. Hong Kong; 13 Multi-Scale Medical Robotics Center, The Chinese University of Hong Kong, Hong Kong, Hong Kong

**Keywords:** MUSE, PROPELLER, DWI, Meningeal lymphatic vessels, Parasagittal dura, Magnetic resonance imaging, Neuroimmunology

## Abstract

The parasagittal dura (PSD) is a thin channel along the sagittal sinus vein at the brain’s upper convexities. Previous studies have shown that cerebrospinal fluid (CSF) flows directly into the PSD, with PSD dimensions and tracer clearance rates associated with aging and brain disorders. Since slow lymphatic drainage is sensitive to water diffusion, PSD circulation may be evaluated using diffusion-weighted imaging (DWI). However, traditional echo-planar DWI (EP-DWI) suffers from low resolution and image distortion, limiting its application to PSD assessment. This study employed high-resolution Multiplexed Sensitivity Encoding (MUSE) DWI and Periodically Rotated Overlapping Parallel Lines with Enhanced Reconstruction (PROPELLER) DWI to investigate PSD water diffusion. These advanced techniques reduce image distortion while enhancing spatial resolution. Our results demonstrated that PSD structures are clearly visible on high-resolution DWI and apparent diffusion coefficient (ADC) maps, correlating with PSD locations identified on T2 FLAIR imaging. In addition, mean ADC values of PSD (1843.1–2062.2 × 10^− 6^ mm^2^/sec) were higher than those of gray and white matter but lower than CSF. These findings highlight the potential of MUSE and PROPELLER DWI for assessing PSD diffusion, offering a promising non-invasive tool for studying PSD circulation and its role in neurological disorders.

**Supplementary Information:**

The online version contains supplementary material available at 10.1038/s41598-025-91751-0.

## Introduction

The lymphatic system is a circulation that contributes to facilitating the clearance of excess fluid and macromolecules from the interstitium. It accomplishes this by passing through lymph nodes to remove bacteria, abnormal cells, and other matter^1^. In brain, the brain glymphatic system (GS)^2^ and the meningeal lymphatic vessels (MLVs)^3^ have been discovered since 2012. The brain GS collects cerebrospinal fluid (CSF) from the subarachnoid space and brain interstitial fluid (ISF) through aquaporin-4 (AQP4) water channels. The MLVs situated in the dorsal and basal regions serve as downstream channels, responsible for draining ISF, macromolecules, and immune cells out of the cranial cavity. Ultimately, the brain GS fluid is drained through the MLVs into the deep cervical lymph nodes^4^. Additionally, they play a crucial role in regulating immune responses in the brain. Recent research has shown that ISF, CSF, MLVs, and the brain GS are important factors impacting brain homeostasis^3^. A numerous studies have reported the association between brain disorders and impaired GS and MLVs, such as cerebral small‑vessel disease^5^, ischemic stroke^6^, and dementia^7^.

Using high-resolution 3D T2-Fluid Attenuated Inversion Recovery (FLAIR) magnetic resonance imaging, dural lymphatic structures were identified along the dural venous sinuses in the dorsal regions and along the cranial nerves in the ventral regions of the human brain^8^. Whole-brain high-resolution 3D T2 FLAIR images showed that the parasagittal dura (PSD) space is the largest region for observing MLVs^9^. The function of parasagittal MLVs and the flux rate of cerebrospinal fluid to the PSD have been evaluated using the propagation of a tracer with multi-phase T1-weighted imaging (T1WI) after the injection of a contrast agent^10^. However, the observation period in multi-phase T1WIs exceeds four hours because of the extremely slow fluid drainage in the PSD space.

The function of PSD can potentially be evaluated using echo-planar diffusion-weighted imaging (EP-DWI) since the slow lymphatic drainage is sensitive to water diffusion. The ability of DWI to assess water mobility makes it a valuable tool for evaluating how well lymphatic drainage functions in these areas, potentially offering diagnostic information for various central nervous system pathologies. Unfortunately, due to the characteristics of low resolution and image distortion^11^, EP-DWI cannot be effectively applied to the PSD. As a result, to our knowledge, diffusion MRI has not yet been directly applied to PSD for diffusion measurement in the literature. In contrast, either multiplexed sensitivity encoding (MUSE) technique^12^ or periodically rotated overlapping parallel lines with enhanced reconstruction (PROPELLER)^13^ can provide diffusion weighted image (DWI) with high spatial resolution and reduced image distortion. In this study, MUSE DWI and PROPELLER DWI with high resolution were used to investigate water diffusion in the PSD space.

## Results

**Fig. 1:**
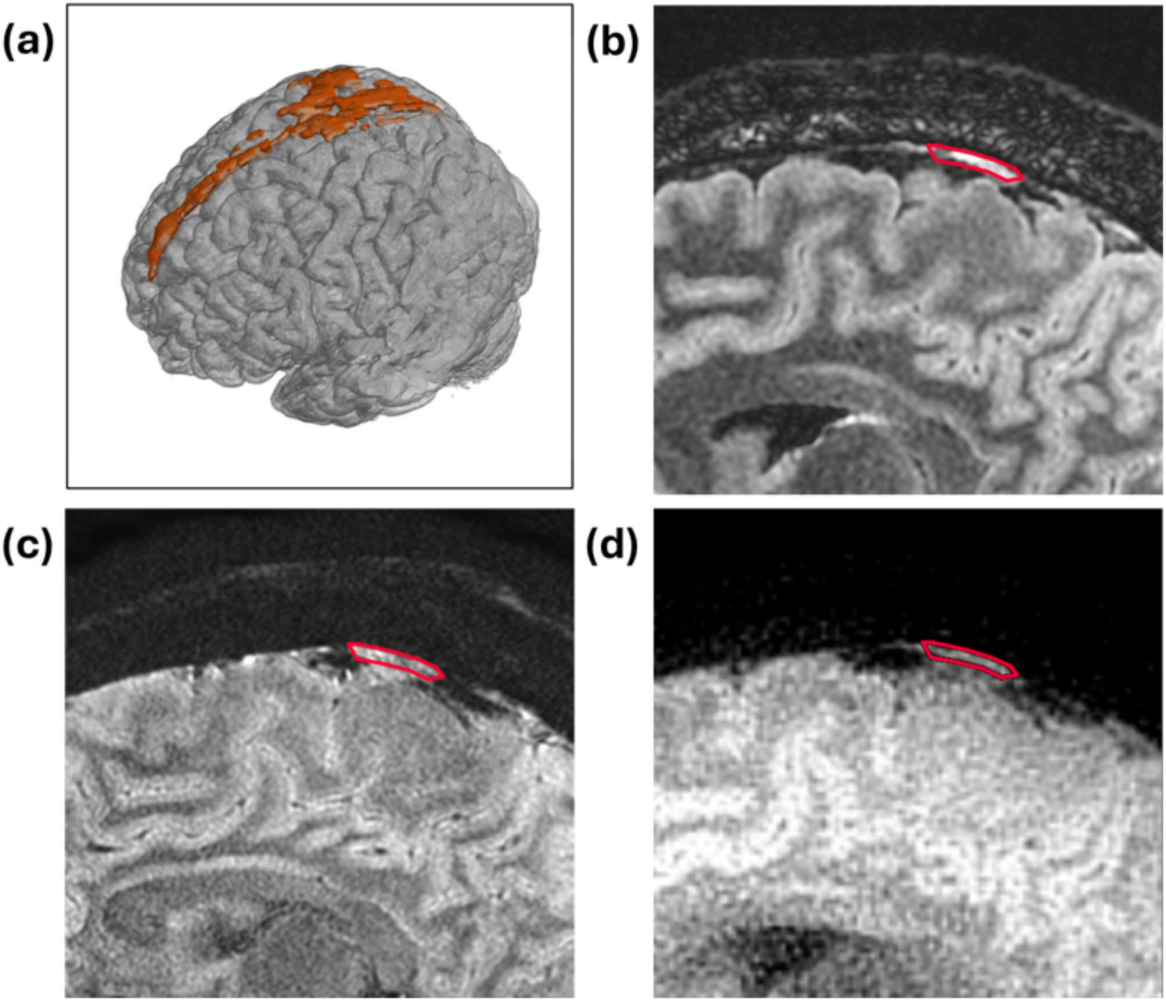
(**a**) 3D FLAIR reconstruction showing the PSD structure in orange, (**b**) 2D T2 FLAIR image, (**c**) MUSE DWI, and (**d**) PROP DWI. The red contour outlines the PSD region, as identified on DWI, corresponding to the high signal in the PSD on FLAIR images. This overlay enhances geometric accuracy visualization and highlights the improved delineation of the PSD structure using MUSE and PROP techniques.

**Fig. 2:**
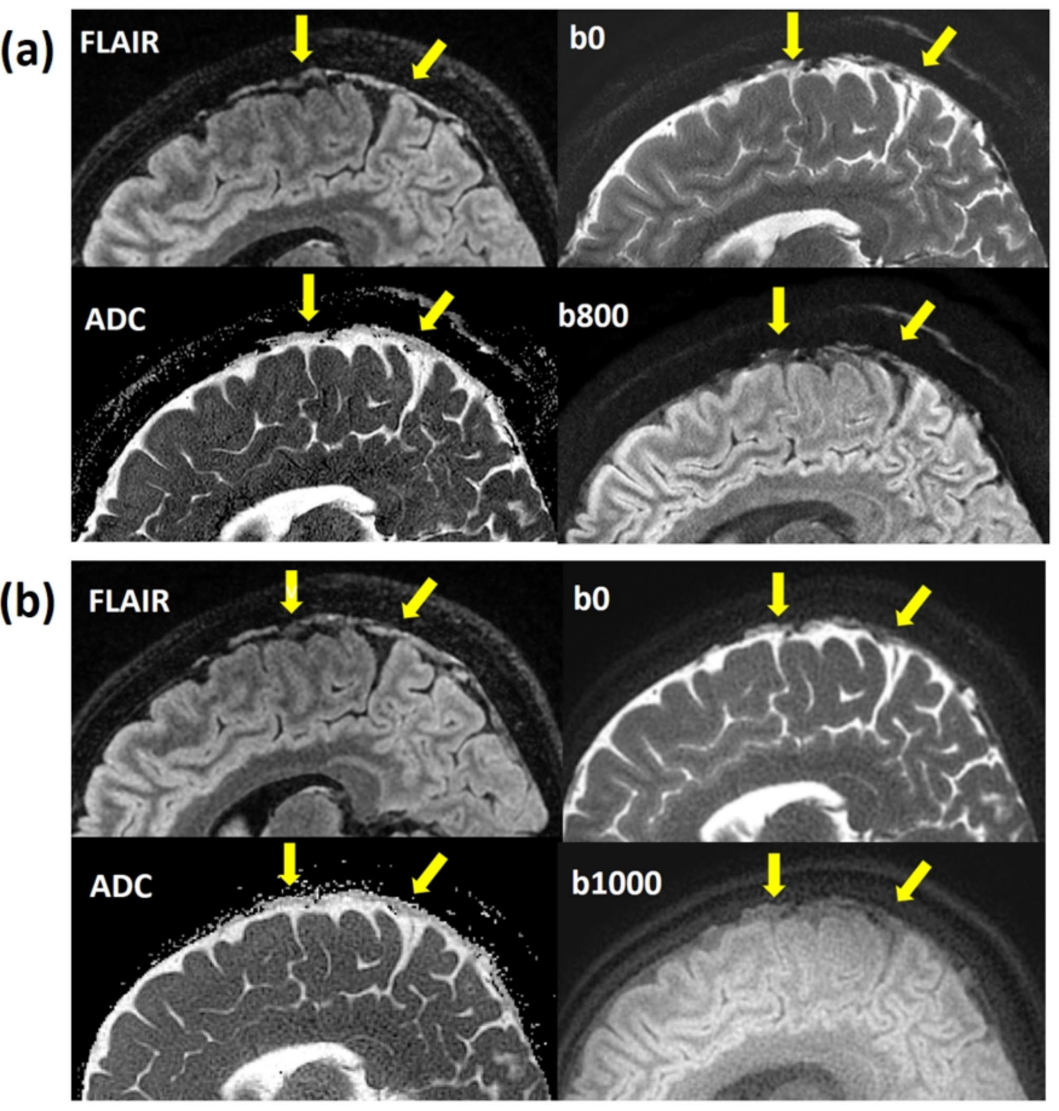
(**a**) The sagittal view displayed the PSD on T2 FLAIR image (up left), MUSE DWI b0 (up right), MUSE DWI b800 (down right), and MUSE ADC map (down left) as indicated by arrows. (**b**) The sagittal view displayed the PSD on T2 FLAIR image (up left), PROPELLER DWI b0 (up right), PROPELLER DWI b1000 (down right), and PROPELLER ADC map (down left) as indicated by arrows. The ADC of the PSD appeared darker than that of the CSF.

**Fig. 3:**
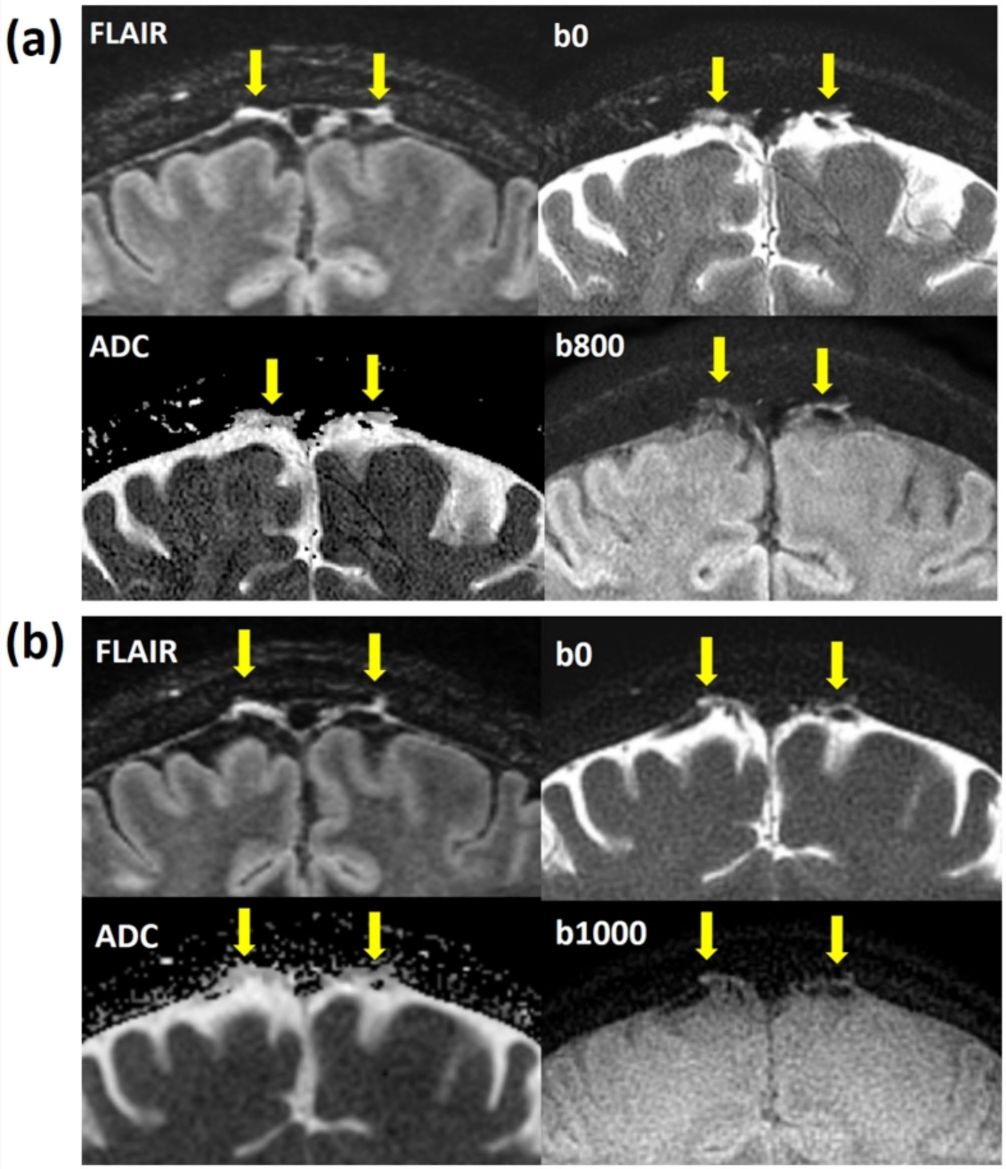
(**a**) The coronal view displayed the PSD on T2 FLAIR image (up left), MUSE DWI b0 (up right), MUSE DWI b800 (down right), and MUSE ADC map (down left) as indicated by arrows. (**b**) The coronal view displayed the PSD on T2 FLAIR image (up left), PROPELLER DWI b0 (up right), PROPELLER DWI b1000 (down right), and PROPELLER ADC map (down left) as indicated by arrows. The ADC of the PSD appeared darker than that of the CSF.

**Fig. 4:**
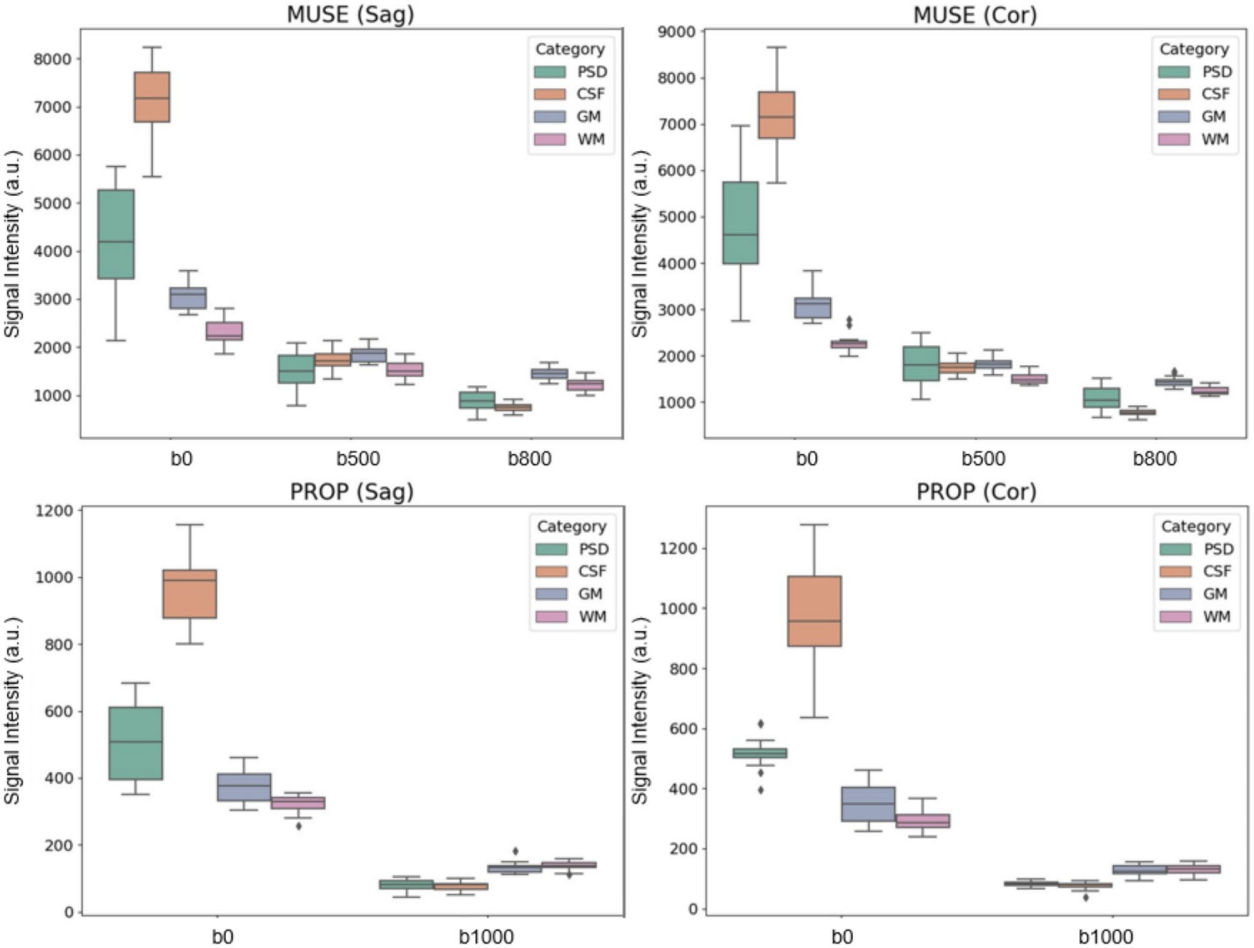
Box plots illustrating the DWI signal intensity of PSD, CSF, GM, and WM across b-values of 0, 500, 800, and 1000 in both sagittal and coronal views. (**a**) MUSE DWI in the sagittal plane, (**b**) MUSE DWI in the coronal plane, (**c**) PROPELLER DWI in the sagittal plane, and (**d**) PROPELLER DWI in the coronal plane.

**Fig. 5:**
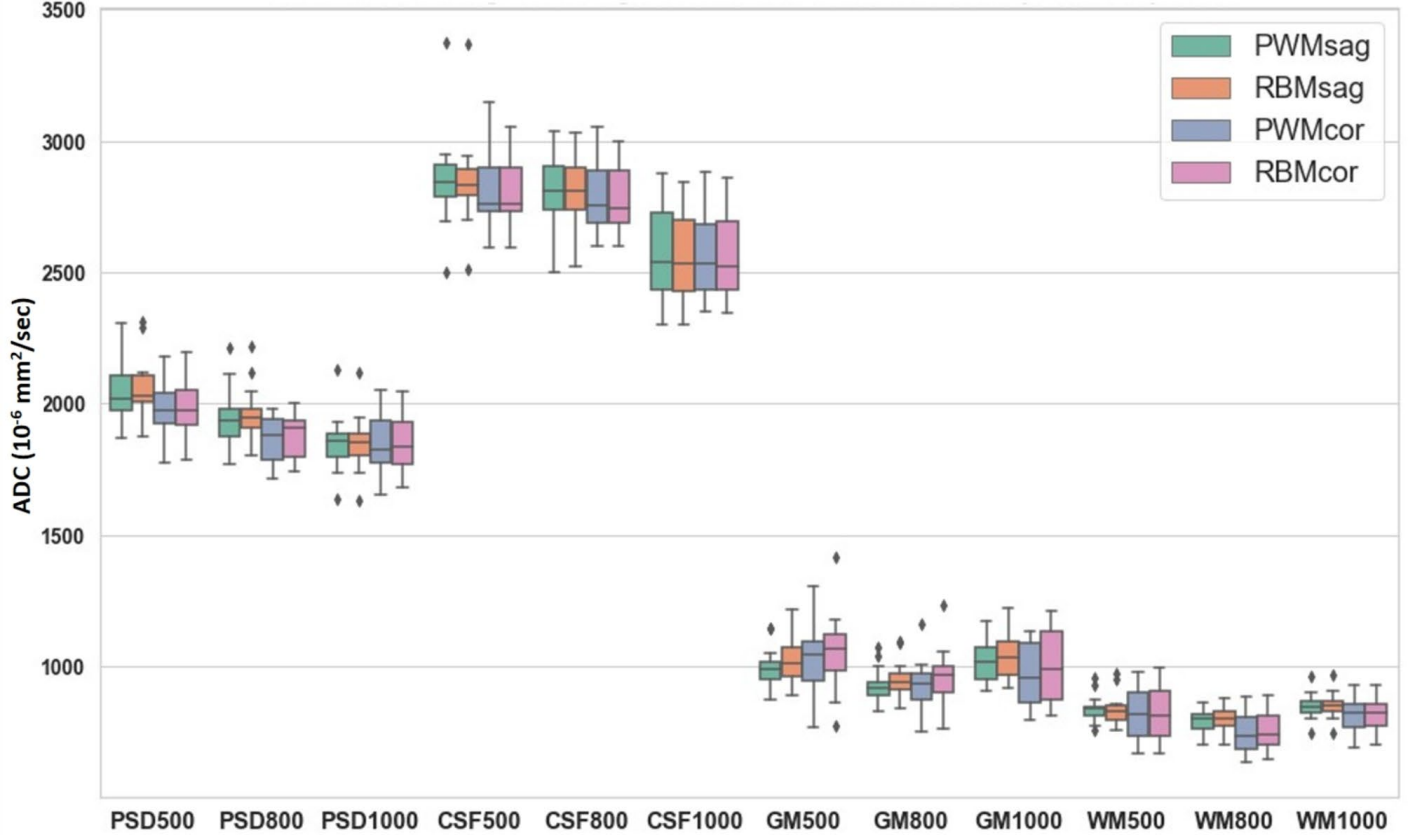
The box plot illustrates the ADC values of PSD, CSF, GM, and WM based on b-values of 500, 800, and 1000, using the PWM and RBM methods in both sagittal (PWMsag, RBMsag) and coronal (PWMcor, RBMcor) views. Each label on the x-axis represents a specific condition: PSD500, PSD800, and PSD1000 represent PSD ADC values measured with b = 0 combined with b = 500 in the MUSE scan, b = 0 combined with b = 800 in the MUSE scan, and b = 0 combined with b = 1000 in the PROPELLER scan, respectively. Similarly, CSF500, CSF800, CSF1000 correspond to CSF measurements; GM500, GM800, GM1000 correspond to GM measurements; and WM500, WM800, WM1000 correspond to WM measurements under the same conditions.

The representative high-resolution T2 FLAIR image and 3D reconstruction of PSD were shown in Fig. 1. The arrows pointed the PSD locations with similar present with previous studies^8^. Figure 2 displayed sagittal view images, including T2 FLAIR, MUSE DWI, PROPELLER DWI, MUSE apparent diffusion coefficient (ADC) map, and PROPELLER ADC map. Figure 3 displayed the coronal view images. Table 1 presented the signal intensity (mean ± SD) of MUSE DWI and PROPELLER DWI for white matter (WM), gray matter (GM), PSD, and CSF in both the sagittal and coronal views. Table 2 listed ADC values (mean ± SD) of WM, GM, PSD, and CSF, measured using the pixel-wise method (PWM) and the ROI-based method (RBM) across sagittal and coronal view. In the study, we compared the ADC values between the different categories PSD, CSF, GM, and WM. This box plot visualized the distribution of DWI intensity and ADC values of WM, GM, PSD, and CSF across PWM and RBM measurements in sagittal and coronal view (Figs. 4 and 5).

**Table 1:** DWI signal intensity (mean  ±  SD) of parasagittal dura (PSD), cerebrospinal fluid (CSF), gray matter (GM), and white matter (WM).

		PSD	CSF	GM	WM
MUSE (Sag)	b0	4202.9 ± 1136.8	7087.3 ± 830.6	3075.3 ± 280.0	2308.0 ± 255.8
	b500	1495.0 ± 390.2	1706.7 ± 218.9	1844.4 ± 153.1	1520.2 ± 163.6
	b800	872.38 ± 217.56	749.62 ± 99.61	1442.92 ± 127.67	1218.52 ± 135.00
MUSE (Cor)	b0	4891.5 ± 1242.7	7185.2 ± 882.9	3103.7 ± 319.7	2278.6 ± 201.7
	b500	1827.2 ± 471.2	1759.1 ± 163.9	1822.2 ± 150.4	1508.9 ± 117.4
	b800	1085.0 ± 274.3	776.1 ± 72.9	1436.8 ± 114.7	1240.3 ± 92.2
PROP (Sag)	b0	502.4 ± 112.9	967.7 ± 112.3	375.9 ± 50.5	321.6 ± 27.1
	b1000	79.1 ± 17.8	76.0 ± 13.8	132.4 ± 17.2	137.3 ± 13.7
PROP (Cor)	b0	517.9 ± 54.0	977.6 ± 163.7	351.0 ± 67.8	293.7 ± 38.9
	b1000	81.5 ± 9.0	75.7 ± 13.1	127.9 ± 17.8	130.5 ± 18.2

*MUSE* multiplexed sensitivity encoding, *PROPELLER* parallel lines with enhanced reconstruction, *DWI* diffusion weighted image, *Sag* sagittal, *Cor* coronal.

**Table 2:** ADC values (mean  ±  SD) of parasagittal dura (PSD), cerebrospinal fluid (CSF), Gray matter (GM), and white matter (WM).

×10^− 6^ mm^2^/sec			PSD	CSF	GM	WM
MUSE ADC (b = 500)	Sag	PWM RBM	2048.7 ± 116.5 2062.2 ± 110.9	2852.6 ± 177.5 2849.8 ± 173.4	991.1 ± 76.9 1021.1 ± 97.3	837.6 ± 52.9 834.4 ± 58.3
	Cor	PWM RBM	1966.1 ± 113.3 1972.3 ± 110.9	2818.8 ± 158.6 2808.2 ± 143.6	1024.1 ± 125.5 1061.8 ± 143.4	821.9 ± 102.4 822.9 ± 105.9
MUSE ADC (b = 800)	Sag	PWM RBM	1942.0 ± 115.7 1957.2 ± 106.3	2810.0 ± 127.4 2810.1 ± 122.1	926.1 ± 65.9 945.7 ± 71.2	789.2 ± 43.1 798.4 ± 45.6
	Cor	PWM RBM	1868.4 ± 84.7 1882.1 ± 84.0	2785.9 ± 138.7 2778.2 ± 133.0	931.7 ± 95.8 960.3 ± 107.7	750.2 ± 77.6 759.0 ± 77.6
PROP ADC (b = 1000)	Sag	PWM RBM	1851.7 ± 105.9 1850.5 ± 103.7	2564.0 ± 178.7 2553.9 ± 169.7	1020.8 ± 76.5 1042.0 ± 91.0	847.8 ± 48.2 852.2 ± 48.8
	Cor	PWM RBM	1843.1 ± 117.9 1849.1 ± 111.5	2568.7 ± 171.9 2561.3 ± 167.1	965.3 ± 123.5 1000.7 ± 141.4	807.1 ± 71.2 812.0 ± 69.5

*MUSE* multiplexed sensitivity encoding, *PROPELLER* parallel lines with enhanced reconstruction, *DWI* diffusion weighted image, *Sag* sagittal, *Cor* coronal.

Figure 4 presented box plots illustrating the DWI signal intensity of PSD, CSF, GM, and WM across b-values of 0, 500, 800, and 1000 in both the sagittal and coronal views. In Fig. 5, the ADC values of PSD measured by b = 0 and b = 500 in the MUSE DWI scan are labeled as PSD500, by b = 0 and b = 800 in the MUSE DWI scan as PSD800, and by b = 0 and b = 1000 in the PROPELLER DWI scan as PSD1000. Similarly, CSF500, CSF800, and CSF1000 correspond to the ADCs of CSF; GM500, GM800, and GM1000 correspond to ADCs of GM; and WM500, WM800, and WM1000 correspond to ADCs of WM produced by two MUSE DWI and one PROPELLER DWI scans. The PWM and RBM measurements appear to have similar distributions across most measures, and Kruskal-Wallis H test suggested consistency between the two methods (*p* > 0.05).

ADC values of PSD gradually decreased with increasing b-values. The mean ADC values were slightly above 2000 × 10^–6^ mm²/sec in PSD500, slightly lower in PSD800, and around 1850 × 10^–6^ mm^2^/sec in PSD1000. Similarly, mean ADC values of CSF showed a gradual decrease from low b-values to high b-values. The ADC values were slightly below 2900 × 10^–6^ mm^2^/sec in CSF500, slightly lower in CSF800, and around 2550 × 10^–6^ mm^2^/sec in CSF1000. However, ADC values of GM and WM decreased from b = 500 to b = 800 in the MUSE scan but slightly increased at b = 1000 in the PROPELLER scan. Mean ADC values of GM were slightly above 1000 × 10^–6^ mm^2^/sec in GM500, slightly below 1000 × 10^–6^ mm^2^/sec in GM800, and returned to around 1000 × 10^–6^ mm^2^/sec in GM1000. Similarly, mean ADC values of WM were slightly above 800 × 10^–6^ mm^2^/sec in WM500, slightly below 800 × 10^–6^ mm^2^/sec in WM800, and returned to above 800 × 10^–6^ mm^2^/sec in WM1000. The Kruskal-Wallis H test and subsequent pairwise comparisons using the Mann-Whitney U test with Dunn–Bonferroni post hoc correction indicated that the ADC of PSD significantly differed from those of CSF, GM, and WM (*p* < 0.001).

## Discussion

In this study, high-resolution 3D T2 FLAIR was used as the standard reference for investigating the feasibility of visualizing the PSD using high-resolution MUSE DWI and PROPELLER DWI. The 3D T2 FLAIR MRI technique is particularly effective at visualizing certain brain structures and pathologies by suppressing the signal from CSF^14–16^. By nullifying the bright CSF signal within the ventricular compartment and subarachnoid space, T2 FLAIR enhances the contrast between the PSD with its high signal and surrounding structures with darker signals, facilitating the differentiation and visualization of small structures such as MLVs and perineural spaces in coronal and sagittal views^10^.

### Utility and limitations of T2 FLAIR imaging for visualizing PSD

Previous studies have utilized high-resolution T2 FLAIR to explore MLVs^10,17^ and arachnoid granulations^18^ in the PSD. Furthermore, T2 FLAIR has been extended to investigate extracranial lymphatic structures from intracranial regions, tracing the pathways of MLVs around venous sinuses and cranial nerves^8^, as well as periarterial fluid drainage along the vessel walls of the internal carotid arteries, which then connect to the cervical lymph nodes^19^. Some findings are corroborated by a study using contrast agent administration^20^. In summary, T2 FLAIR’s ability to suppress CSF signals, enhance contrast, and provide high-resolution images makes it an excellent tool for visualizing MLVs and related drainage pathways in the brain. Additionally, T2 FLAIR provides high-resolution images that are essential for evaluating PSD thickness in aging^8^ and PSD volume in various CSF disorders^21^. The benefits of T2 FLAIR MRI include its noninvasive nature and the lack of need for contrast agents, making it suitable for patients who cannot receive contrast media and enabling longitudinal studies. However, although high-resolution FLAIR can clearly depict the PSD structure, it only provides limited information on functional and pathological characteristics.

### Challenges and advances in DWI techniques for PSD imaging

The PSD is located on both sides of the superior sagittal sinus and contains abundant arachnoid granulations and parasagittal MLVs^21^. These vessels play a crucial role in draining interstitial fluids out of the brain and contributing to the glymphatic system^22^. In this study, we evaluated the feasibility of measuring the ADC of the PSD using high-resolution MUSE DWI and PROPELLER DWI. Single-shot EP-DWI (SS-EP-DWI) is the most widely used DWI sequence in clinical practice, with typical voxel sizes around (1.5—1.8)×(1.5—1.8)×(5—6) mm^3^ in routine SS-EP-DWI. However, SS-EP-DWI is prone to high geometric distortion and low spatial resolution due to its sensitivity to magnetic susceptibility artifacts, especially near air and bone interface^11^. For example, the geometric distortion of the parotid glands can result in body surface length errors of 7–12%^23^, affecting 27–30% of a population^24^. The PSD, like the parotid glands, is located at brain convexities near the skull, making it susceptible to geometric distortion from magnetic susceptibility artifacts. Additionally, MLVs are predominantly located in the PSD^22^, and the dura mater is a flimsy structure with varying thickness. One study reported a mean thickness ranging from 0.35 mm to 1.1 mm^25^, while the other measured a mean thickness of 1.14 ± 0.06 mm^26^. Consequently, the PSD space may be missed in SS-EP-DWI due to image distortion and low spatial resolution, highlighting the need for DWI techniques with less distortion and higher resolution to accurately observe the PSD space.

Numerous studies have indicated that MUSE DWI and PROPELLER DWI outperform SS-EP-DWI by providing less distorted images and higher spatial resolution, as both are multi-shot diffusion-weighted imaging techniques based on different principles. MUSE DWI has been proposed to improve spatial resolution and geometric fidelity in brain DWI^27^, while PROPELLER DWI offers better quality in terms of distortion, susceptibility-related changes, and lesion conspicuity^28^. Our results showed that the PSD space was clearly visible on both MUSE and PROPELLER imaging with high-resolution DWI and their ADC maps, corresponding to the T2 FLAIR image in both sagittal and coronal views. In this study, MUSE DWI with a voxel size of 0.65 × 0.65 × 3 mm^3^ and PROPELLER DWI with a voxel size of 0.78 × 0.78 × 3 mm^3^ were used to evaluate water diffusion in the PSD space. Therefore, high spatial resolution DWI with minimal distortion is important for accurate ADC measurement in the PSD. Note that further reduction of geometric distortion or improvement of spatial resolution in MUSE DWI may necessitate using a higher number of shots (e.g., eight shots or more), but the maximum achievable number of shots for MUSE DWI is typically limited by the availability of receiver channels. On the other hand, although PROPELLER DWI with fast-spin echo readout can produce distortion-free images, it is generally less scan-efficient compared to MUSE DWI (two b-values versus three b-values acquired with comparable scan time). The better scan efficiency of MUSE DWI may allow for ADC measurement with multiple b-values. Nevertheless, PROPELLER DWI is less susceptible to head motions (e.g., head tremor) and may be more suitable for assessing PSD space in challenging subjects. Ultimately, the choice between the two techniques may depend on their availability on the MRI scanner, the desired number of b-values, and the target patient populations.

### Role of ADC in characterizing water diffusion in the PSD

ADC is a quantitative metric that reflects the microscopic environment by measuring the diffusibility of water in brain tissue. ADC plays an important role in diagnosing diseases that affect various tissue types and involve different pathological processes, such as ischemic stroke, neoplasms, intracranial infections, traumatic brain injury, and demyelinating conditions^29^. Our study showed that the ADC values in the PSD space are intermediate between those of brain parenchyma (GM and WM) and CSF, as diffusion is more restricted in bound water, such as within solid tissue or macromolecules. The protein-rich fluid, which includes waste or macromolecules draining from the CSF-ISF-CSF washout process originating from the perivenous space, eventually drains into the PSD, which has a thin film space^30^. Consequently, the concentration of macromolecules in the PSD is higher than in CSF due to macromolecular accumulation and the narrow space within the PSD. Therefore, our study revealed that ADCs in the PSD are higher than in solid tissues like GM and WM but lower than in the free fluid of CSF.

### Controversies and advancements in PSD imaging: insights into neuroimmune function and circulation efficiency

Some studies refer to the bright signal region in the meninges on T2 FLAIR images as MLVs^8,10,17^. However, other research challenges this interpretation^31,32^. The primary contradictions arise from the fact that hyperintense FLAIR signals in the PSD may be attributed not only to lymphatic tissue but also to other tissues. Additionally, MLVs may be influenced by partial volume effects, as they are often too small to be reliably identified at a 1 mm spatial resolution on T2 FLAIR images. Although the identification of a lymphatic signal in the PSD on FLAIR MRI remains heavily contested^31–34^, there is strong evidence suggesting that the PSD serves as a potential neuroimmune interface. Numerous immune-related tissues and brain metabolites have been found within the PSD space^18,35^. Dural channels within the PSD also act as reservoirs for cerebrospinal fluid drainage^36^. Moreover, the rate of contrast agent wash-in and wash-out in the PSD is influenced by factors such as aging^22,37^, brain parenchymal fraction^38^, and brain diseases^5,39^. Increasingly, studies reveal that the PSD harbors rich immune elements and that circulation within the PSD is connected to the efficiency of brain waste metabolism and immune function. However, most of these studies rely on contrast agent administration, a procedure that requires several hours to measure the accumulation and drainage of the contrast agent. Our study demonstrated that ADC measurements in the PSD have potential as a rapid metric for assessing PSD circulation efficiency, which can be completed in a few minutes.

### Limitations

Although our study successfully demonstrated the feasibility of measuring PSD ADC using multi-shot high-resolution DWI techniques and confirmed its distinctiveness from WM, GM, and CSF in healthy young subjects, further investigation is needed to enhance clinical applicability. First, the sample size was small, and all subjects were healthy and young, with a mean age of 23 years. While this group was sufficient for testing the reliability of ADC measurements in the PSD, it may not be representative of the broader population.

Second, the use of 2D MUSE DWI and PROPELLER DWI with a 3 mm slice thickness may introduce partial volume effects in the ADC measurements of the PSD. This challenge arose from both the flimsy and fragmented structure of the PSD with varying thickness^25,26^ and the inherent slice thickness limitation of 2D DWI. However, this was a necessary trade-off between slice thickness and sufficient signal-to-noise ratio for 2D acquisition. While we mitigated partial volume contamination by measuring ADC only in thicker PSD regions, this also limited our ability to trace ADC variations across the PSD along the sagittal sinus vein. To address this limitation, future studies could consider using 3D MUSE DWI for more precise PSD ADC measurement^40^, potentially minimizing partial volume effects and achieving higher SNR.

Third, we tested two sequences (MUSE DWI and PROPELLER DWI) and three b-values (500 and 800 for MUSE DWI, and 1000 for PROPELLER DWI) in this study. SNR analysis revealed that high b-values resulted in low SNR in CSF due to fast flow. Although increasing NEX could improve SNR, it would also lengthen the already extensive scan time (over 60 min per participant for acquiring high-resolution DWI with different b-values and scan planes). Therefore, we used b = 800 for MUSE DWI to optimize the trade-off between SNR and scan efficiency. As a result, we could not evaluate the variability of PSD ADC sensitivity across different b-values. Although evaluating PSD ADC variability with multiple b-values is important, this remains an area for future research. Recent studies have explored the intravoxel incoherent motion (IVIM), a DWI technique with multiple b-values, in brain disorders^41^, and our findings suggest its potential for PSD IVIM measurement.

Fourth, our study only showed that PSD ADC values differ from those of other brain tissues (WM, GM, and CSF) previously established in healthy young subjects. Recent studies have explored PSD and MLV morphology and drainage in brain disorders and aging^5,8,21^, revealing variations in volume and lymphatic drainage patterns in pathological conditions. Future studies should validate the reliability and sensitivity of PSD ADC measurements in larger populations, particularly in aging and specific brain disorders.

Additionally, our study did not account for pulsation effects on ADC measurements in the PSD caused by surrounding veins and CSF. Future research should investigate potential biases in PSD ADC values resulting from pulsation effects due to vein and CSF contamination, using techniques such as FLAIR-DWI^42^ and cardiac gating^43^.

## Conclusions

The study successfully demonstrated the feasibility of assessing ADC measurements in the PSD using MUSE DWI and PROPELLER DWI, both of which offer high spatial resolution and minimal image distortion. The high resolution and distortion insensitivity of MUSE DWI and PROPELLER DWI enable the visualization of PSD regions corresponding to their locations on high-resolution T2 FLAIR MRI, supporting the feasibility of measuring diffusion in the PSD in both sagittal and coronal views. Additionally, the ADC values in the PSD are higher than those in GM and WM, but lower than in CSF. The ADC values are consistent whether using the PWM or RBM measurement methods.

## Materials and methods

### Study participants

This study recruited 6 male and 2 female volunteers with a mean age of 23 years (range: 20 to 28 years) who did not exhibit any symptoms related to brain or cerebral circulation. This prospective study was approved by the local institutional review board at China Medical University Hospital (CMUH111-REC1-033). All methods were performed in accordance with relevant guidelines and regulations, and written informed consent was obtained from all participants.

### MRI scans

MR studies were conducted using 3.0T scanners (SIGNA Architect, GE Healthcare) with head and neck coils. A high-resolution 3D T2-FLAIR sequence was applied to locate the PSD, with imaging parameters as follows: repetition time (TR) of 10,000 ms, echo time (TE) of 156 to 160 ms, inversion time (TI) of 2,350 to 2,410 ms, field of view (FOV) of 190 to 200 × 190 to 200 mm, matrix size of 224 × 224, number of excitations (NEX) of 1, and slice thickness of 1 mm. MUSE DWI was performed with 4 shots, using scanning parameters as follows: TR of 5,000 ms, TE of 91 to 95 ms, b-values of 0, 500, and 800 s/mm^2^, 2 NEXs (b = 0), 3 NEXs (b = 500), and 6 NEXs (b = 800), FOV of 190 to 200 × 190 to 200 mm, matrix size of 288 to 320 × 288 to 320, and slice thickness of 3 mm. PROPELLER DWI was also performed, using scanning parameters as follows: TR of 5,000 ms, TE of 91 to 95 ms, ETL 32, b-values of 0 and 1000 s/mm^2^, 2 NEXs (b = 0), and 6 NEXs (b = 1000), FOV of 190 to 200 × 190 to 200 mm, matrix size of 256 × 256, and slice thickness of 3 mm. The protocol parameters for the 3D T2-FLAIR, MUSE DWI, and PROPELLER DWI sequences are summarized in Table 3.

**Table 3:** The protocol parameters for T2 FLAIR, MUSE DWI, and PROPELLER DWI sequences.

	T2 FLAIR (Sag.)	MUSE DWI (Sag.)	PROPELLER DWI (Sag.)	MUSE DWI (Cor.)	PROPELLER DWI (Cor.)
TR	10,000 ms	5000 ms	5000 ms	5000 ms	5000 ms
TE	157 ms	95 ms	81 ms	95 ms	81 ms
TI	2358 ms	NA	NA	NA	NA
b value	NA	0, 500, 800	0,1000	0, 500, 800	0, 1000
number of shots	NA	4	NA	4	NA
NEX	1	2 (b = 0) 3 (b = 500) 6 (b = 800)	1.5 (b = 0) 6 (b = 1000)	2 (b = 0) 3 (b = 500) 6 (b = 800)	1.5 (b = 0) 6 (b = 1000)
FOV	190 × 190 mm	200 × 200 mm	200 × 200 mm	190 × 190 mm	190 × 190 mm
Matrix Size	224 × 224	320 × 320	256 × 256	288 × 288	256 × 256
Slice Thickness	1 mm	3 mm	3 mm	3 mm	3 mm
Scan Time	9:44	10:20	9:50	10:20	9:50

*MUSE* multiplexed sensitivity encoding, *PROPELLER* parallel lines with enhanced reconstruction, *DWI* diffusion weighted image, *Sag* sagittal, *Cor* coronal.

The total scan time was approximately 60–70 min, including the localizer scan, 3D high-resolution FLAIR scan, high-resolution 2D MUSE DWI and 2D PROPELLER DWI in both the sagittal and coronal planes, as well as the reconstruction of three-plane FLAIR images (coronal, sagittal, and axial) for PSD slice localization. These high-resolution 2D DWI scans, consisting of 12–15 slices in the sagittal and coronal planes, were positioned as perpendicular as possible to the PSD channel, targeting clear and thicker PSD regions, based on the three-plane FLAIR images.

### Data processing and analysis

Apparent diffusion coefficient (ADC) maps were generated through pixel-by-pixel computation from DWI images using the Stejskal–Tanner formula: $\:SI\left(b\right)=\:SI\left(b=0\right)\bullet\:{e}^{-b\bullet\:ADC}$. ADC values were measured within regions of interest (ROI) encompassing four tissues: the PSD, gray matter (GM), white matter (WM), and CSF. ROIs on the ADC map, corresponding to areas on FLAIR images, were placed to avoid partial volume effects and extra-tissue noise. The ROIs were drawn by a neuroradiologist with 3 years of experience in neuroradiology. For each subject, two ROIs with clear and sufficiently large regions for PSD, CSF, GM, and WM were selected in both sagittal and coronal slices. The mean ADC values were generated using both a pixel-wise method (PWM) and an ROI-based method (RBM) in sagittal and coronal views for comparison, denoted as PWMsag, RBMsag, PWMcor, and RBMcor. Finally, the mean values and standard deviations for the four tissues across all subjects were calculated for analysis. The signal-to-noise ratio (SNR) analysis was conducted using the dual acquisition (subtraction) technique in the study. Detailed methods and results are provided in the supplementary information.

### Statistical analysis

Statistical analysis was performed by using SPSS software (IBM SPSS Statistics for Windows, Version 24.0; IBM Corp., Armonk, NY). The Kruskal-Wallis H test was used to compare the four groups (PWMsag, RBMsag, PWMcor, RBMcor) across the different categories (e.g., PSD, CSF, GM, WM), and significant difference among the four tissue types (PSD, CSF, GM, WM). When the significant difference in Kruskal-Wallis H test was found in the group comparison, then Mann-Whitney U test applying a Dunn–Bonferroni post hoc correction was used to compare each pair. A *p* value less than 0.05 was considered to indicate statistical significance.

## Electronic supplementary material

Below is the link to the electronic supplementary material.

Supplementary Material 1

## Data Availability

The data analyzed during the current study are available from the corresponding author on reasonable request.

## Abbreviations

PSD: Parasagittal dura
GS: Glymphatic system
ISF: Interstitial fluid
MLV: Meningeal lymphatic vessel
MUSE: Multiplexed sensitivity encoding
PROPELLER: Periodically rotated overlapping parallel lines with enhanced reconstruction
