# Supplementary material for: MUSE and PROPELLER DWI for ADC in parasagittal dura: insights from high-resolution and reduced-distortion DWI
Source: Sci Rep. 2025 Mar 3;15:7473. doi: 10.1038/s41598-025-91751-0 (PMC11876650; doi:10.1038/s41598-025-91751-0)
Supplement: Supplementary file 1 — Supplementary Material 1 [file 41598_2025_91751_MOESM1_ESM.docx]

Signal-to-noise ratio Analysis

The conventional signal-to-noise ratio (SNR) analysis is by comparing the average intensity of the region of interest (ROI) to the standard deviation in the background or air region. However, the MUSE DWI and PROPELLER DWI images are reconstructed using algorithms provided by the MRI manufacturer. Consequently, the signal from air outside the brain is processed and does not accurately represent background noise (Figure S1). To address this limitation, SNR analysis was performed using the dual acquisition (subtraction) technique [1].

**Materials and Methods**

Three participants were recalled for dual MRI acquisition under the same MRI scanner and protocols used in the main study. Each participant underwent two consecutive measurements for MUSE DWI and PROPELLER DWI. The SNR was calculated using the dual acquisition subtraction method with the following formula:

SNR = $\sqrt{2} \mu_{signal}/\sigma_{diff}$

Where: 𝜇_signal_ is mean intensity of the signal region (ROI) from the first acquisition, and 𝜎_diff_ is the standard deviation of the corresponding ROI in the difference image (obtained by subtracting the two acquisitions). For each subject, two ROIs with clear and sufficiently large regions for PSD, CSF, GM, and WM were selected on both sagittal and coronal slices for MUSE DWI and PROP DWI, then calculated the mean intensity, SD, and SNRs in MUSE DWI (b0, b500, and b800) and PROP DWI (b0 and b1000).

**Results**

Figure S2 showed the MUSE DWIs at b=0, 500, and 800, along with their corresponding difference images. Figure S3 presented the box plots illustrating signal intensity, standard deviation, and SNR. In the figure, measurements for MUSE DWI at b=0, b=500, and b=800 are labeled as MUSE0, MUSE500, and MUSE800, respectively. Similarly, PROP0 and PROP1000 correspond to PROPELLER DWI at b=0 and b=1000. Table S1 provided the mean ± SD values for signal intensity, standard deviation, and SNR across the different tissue categories (PSD, CSF, GM, and WM).

**Discussion**

Unlike conventional SNR estimation methods, the dual acquisition subtraction technique does not rely on background noise measurements. Instead, it directly quantifies noise by computing the difference between two consecutive acquisitions, making it more robust and reliable. This approach is particularly useful when measuring noise in challenging conditions where background noise is difficult to assess.

Due to the lengthy 1.5-hour scan time required for high-resolution MUSE DWI and PROPELLER DWI, only three out of eight participants who were stable and able to tolerate long scan durations were included in this analysis.

Our results demonstrate that the mean SNR ranged from 7 to 20 in both MUSE and PROPELLER DWI across PSD, CSF, GM, and WM. The SNR at b=0 was comparable between the two techniques. Although the observed SNR values were lower than those in conventional DWI due to high resolution, thin slice thickness, long scan times, and different SNR calculation, they were still sufficient to differentiate PSD ADC values from CSF, GM, and WM.

Reference

1. Firbank MJ, Coulthard A, Harrison RM, Williams ED. A comparison of two methods for measuring the signal to noise ratio on MR images. Phys Med Biol. 1999 Dec;44(12):N261-4. doi: 10.1088/0031-9155/44/12/403. PMID: 10616158.

Table S1. The values (mean ± SD) of the signal intensity, standard deviation, and SNR in parasagittal dura (PSD), cerebrospinal fluid (CSF), gray matter (GM), and white matter (WM) across different b-values.

|  |  |  | PSD | CSF | GM | WM |
| --- | --- | --- | --- | --- | --- | --- |
| Signal Intensity | MUSE | b0  b500 | 4319.0±901.8  1597.2±346.0 | 7126.9±661.0  1684.3±224.2 | 2696.7±217.5  1670.0±74.6 | 1963.1±101.8  1315.5±78.9 |
|  |  | b800 | 962.3±188.4 | 755.6±107.9 | 1328.2±27.8 | 1067.5±69.1 |
|  | PROP | b0  b1000 | 492.6±131.3  75.4±14.7 | 868.3±81.4  71.4±10.9 | 311.9±46.1  110.0±11.1 | 270.2±36.7  113.9±10.5 |
| Standard Deviation | MUSE | b0  b500 | 522.3±132.1  169.7±43.8 | 563.4±212.0  180.0±40.4 | 407.2±79.5  161.5±20.2 | 359.3±38.9  159.2±18.9 |
|  |  | b800 | 107.4±33.1 | 102.3±22.5 | 121.5±28.8 | 114.1±17.6 |
|  | PROP | b0  b1000 | 67.2±11.8  14.8±5.4 | 63.9±15.0  15.0±2.7 | 55.1±7.5  14.0±1.9 | 49.2±6.8  14.2±2.5 |
| SNR | MUSE | b0  b500 | 12.3±4.2  14.4±5.6 | 19.9±6.1  14.0±4.1 | 9.8±2.4  14.8±2.0 | 7.8±0.9  11.8±1.5 |
|  |  | b800 | 13.5±3.6 | 11.0±3.2 | 16.1±3.0 | 13.6±2.6 |
|  | PROP | b0  b1000 | 10.5±3.0  7.8±2.3 | 20.1±5.1  7.0±1.8 | 8.1±1.5  11.4±2.3 | 7.9±1.9  11.7±2.6 |

MUSE: multiplexed sensitivity encoding; PROPELLER: parallel lines with enhanced reconstruction; SNR: signal-to-noise ratio.


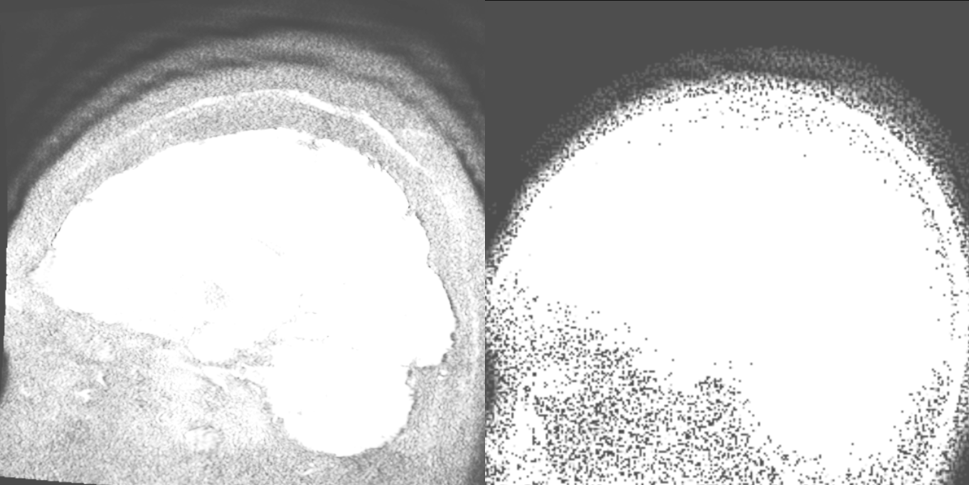
Figures

Figure S1. The images illustrate how background air signals are processed by the MRI reconstruction algorithm.


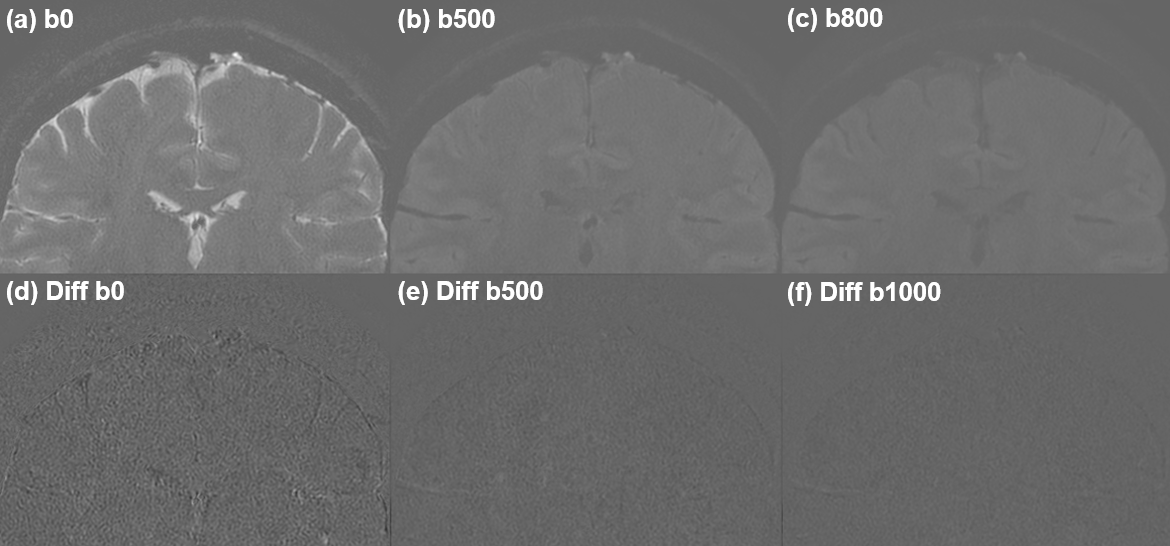


Figure S2. MUSE DWI images at (a) b=0, (b) b=500, and (c) b=800. Their corresponding difference images from two acquisitions are shown in (d), (e), and (f).


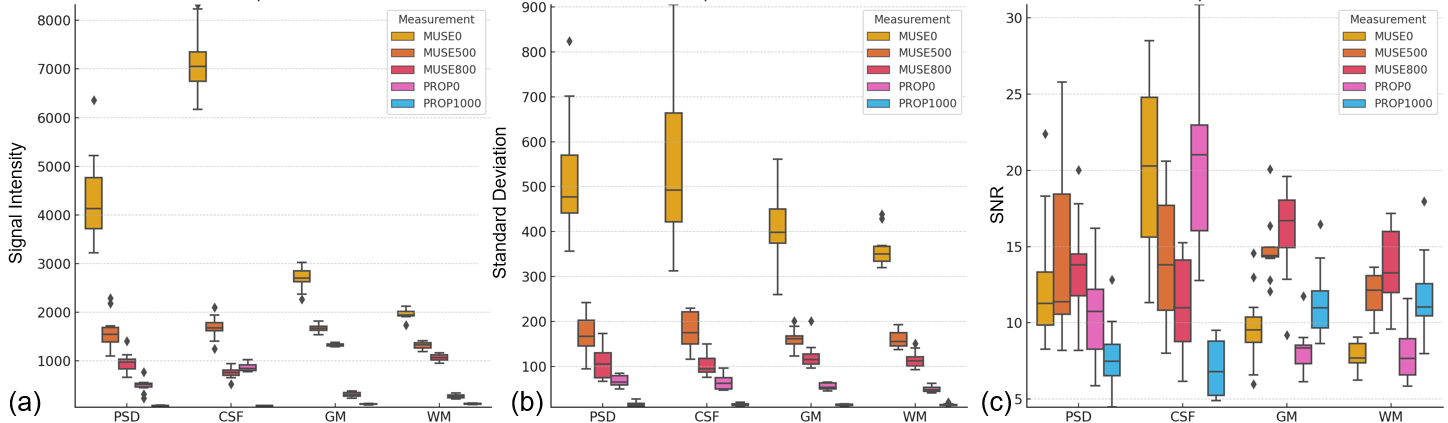


Figure S3. Box plots displaying (a) signal intensity, (b) standard deviation, and (c) SNR. The colored boxes represent MUSE0, MUSE500, and MUSE800 for MUSE DWI at b=0, 500, and 800, respectively, while PROP0 and PROP1000 correspond to PROPELLER DWI at b=0 and 1000.
